# Biomimetic Groundwork for Thermal Exchange Structures Inspired by Plant Leaf Design

**DOI:** 10.3390/biomimetics4040075

**Published:** 2019-11-27

**Authors:** Ariana I. K. S. Rupp, Petra Gruber

**Affiliations:** 1 Department of Biology, Biomimicry Research and Innovation Center, The University of Akron, Akron, OH 44325, USA; 2 Myers School of Art and Department of Biology, Biomimicry Research and Innovation Center, The University of Akron, Akron, OH 44325, USA; pgruber@uakron.edu

**Keywords:** leaf morphology, heat exchange, thermal design, bio-inspired structures, evaporation, shape analysis

## Abstract

Geometry is a determining factor for thermal performance in both biological and technical systems. While biology has inspired thermal design before, biomimetic translation of leaf morphology into structural aspects of heat exchangers remains largely unaddressed. One determinant of plant thermal endurance against environmental exposure is leaf shape, which modulates the leaf boundary layer, transpiration, evaporative cooling, and convective exchange. Here, we lay the research groundwork for the extraction of design principles from leaf shape relations to heat and mass transfer. Leaf role models were identified from an extensive literature review on environmentally sensitive morphology patterns and shape-dependent exchange. Addressing canopy sun–shade dimorphism, sun leaves collected from multiple oak species exceeded significantly in margin extension and shape dissection. Abstracted geometries (i.e., elongated; with finely toothed edges; with few large-scale teeth) were explored with paper models of the same surface area in a controlled environment of minimal airflow, which is more likely to induce leaf thermal stress. For two model characteristic dimensions, evaporation rates were significantly faster for the dissected geometries. Shape-driven transfer enhancements were higher for the smaller models, and finely toothed edges reached local cooling up to 10 °C below air temperature. This investigation breaks new ground for solution-based biomimetics to inform the design of evaporation-assisted and passively enhanced thermal systems.

## 1. Introduction

### 1.1. Thermal Design Innovation and Biomimetics

Technical systems for heat transfer are necessary and widespread in a variety of realms, from everyday objects to industry to architecture. Generally speaking, the multifold repercussions of thermodynamics justify current thermal engineering and design efforts invested in all sorts of systems. With devices becoming increasingly compact and powerful, heat dissipation is an escalating problem for product development, even more so in miniaturized electronics [1]. While integration of liquids brings design difficulties, especially in small and electrical products, evaporative phase-change media are cost effective and often needed in up-to-date thermal technology. The market for fluid-assisted thermal exchangers is long standing, still exploiting self-contained evaporative phase-change (e.g., heat pipes, cold plates) and benefiting from design innovation [2,3]. That is because spatial configuration is a fundamental aspect of thermal design, and passive enhancement of transfer can be achieved via only making geometry adjustments [4].

Thermal engineering literature attests to the importance of geometry, addressing its complex effects on a case study basis (i.e., idealized geometries, such as the flat plate, infinite pipe, and sphere cases). Space variables—scale, surface area to volume ratio, overall shape, interface geometry, texture, roughness, orientation, inclination, arrangement—can affect heat transfer to different degrees. However, the process for thermal design innovation, namely the development of more intricate structures, is challenging. For atypical geometries, analytical solutions are not always possible to derive and the use of numerical methods [5] is often too limited for complex phenomena, such as three-dimensional convective transfer combined with phase change [6]. In those cases, geometry for enhanced transfer may be explored with reverse-engineering thinking and iterative experimental testing of design assumptions, rather than being deduced from mathematical optimization or computational simulation [7]. Here, we propose biomimetics—biological insight taken into the technical realm—to provide an alternative workflow for design innovation to the long-established prescriptive protocols framing thermal engineering practice.

With the advent of thermal biology, principles from physics and thermodynamics have been applied in an increasingly systematic and meticulous manner to explain how organisms remain and thrive within specific temperature ranges, despite their continuously fluctuating environment. Biologists have demonstrated the existence of functional, adaptive thermal features, which can be of a structural, physiological, or behavioral nature [8], and inform thermal technology. The potential of thermal biomimetics is illustrated by nature-inspired insulation materials [9,10,11], passive ventilation techniques (Harare Eastgate Centre building in Zimbabwe [12]), energy management algorithms for climate control equipment (i.e., Encycle Swarm Logic^®^ [13]), vascular cooling design for injection molds [14] or solar panels [15], responsive architectural façades [16], and evaporation-driven micropumps for drug delivery [17].

### 1.2. Plant Structures, Leaf Exchange, and Thermodynamics

Besides serving as solar power stations for the plant, leaves are persistent heat exchangers and water vapor dissipators. Regarding heat exchange, exposure to the environment, seasons, and sunlight can shift leaf temperatures within a range of 50 °C [18]. As any heated surface, a leaf above air temperature will dissipate heat by different mechanisms and to different degrees, depending both on leaf and environmental properties. Typical modes of heat transfer are net radiation resulting from solar and environmental inputs minus the leaf’s radiative heat loss, heat conduction through the ground, heat convection through air, and evapotranspiration [19]. Regarding mass exchange, a plant loses about 97% of its water intake through the leaves [20], which continuously undergo passive and metabolic mechanisms of water management. When a leaf transpires, it introduces additional modes of heat transfer, such as evaporative cooling, also affecting leaf temperature to different degrees. Most importantly, leaves may thermally decouple from the environment, avoiding overheating and reaching temperatures more favorable to photosynthetic metabolism. For these reasons, plant thermal management and limited homeothermy is acknowledged among botanists [21,22]. Hence, leaves provide a biological role model for heat transfer assisted by fluid phase change.

Disregarding the complexity of physiology and behavior, the opportunity for structural learnings taken from biology and applied to heat transfer is promising. Structural strategies and information management have been identified as nature’s recurrent pathways to problem solving, in contrast to human technology’s preference for energy manipulation [23]. Unique structures are found in plants, specifically, given their limited physiology and behavior. For instance, biomimetic innovation breakthroughs have originated from leaf microstructures (e.g., Lotus effect [24], pitcher plant ultra-slippery surfaces [25], Salvinia effect [26]). While leaf optical properties, photosynthesis energetics, venation design, and tissue micromorphology are inspiring up-to-date research, leaf blade overall shape and potential effects on convective transfer remain unaddressed.

Based on the theory that leaf design significance lies between hydraulic and thermal management [27], we propose the interpretation of leaves as heat and mass exchange biostructures. This article reviews the literature on leaf transfer and morphology, identifies role models and design hypotheses of interest, exposes knowledge gaps about the relation between shape and transfer, tests simple geometry parameters as a first approximation to such a relation, and delivers some first experimental results. By revisiting botany under the exchanger design lens, here, we lay the groundwork for the extraction of geometrical principles from leaves’ rich and miscellaneous shape portfolio.

## 2. Materials and Methods

The biomimetic process has been described to be either problem or solution based [28]. Even though the general problem area of evaporative thermal devices is targeted, the research method undertaken here rather follows a solution-based approach, with biological role models identified in advance. This orients research efforts towards the basic bioscience of a subject not addressed yet—leaf-inspired thermal exchangers—and exploration of the biological solution. Principles extracted will be reframed and applied to the technical problem in future stages of the research.

The following steps, specifically, framed biology search, research, and biomimetic abstraction:

- Literature review and identification of botanical case studies pointing to a relation between leaf thermal function and morphology patterns—listed as leaf role models;
- Definition of leaf morphotypes and shape features of interest involved in such relations, presumed relevant for leaf exchange and plant thermal management, based on botany and transfer physics literature;
- Identification of thermal design features and hypotheses abstracted from the leaf literature review—listed as leaf-inspired design principles;
- Biology research addressing one instance of the reviewed case studies, that of sun–shade leaf dimorphism in oak trees. The experimental approach involved shape analysis of oak leaves with single-parameter metrics. This tested the suitability of basic geometrical parameters for differentiating sun and shade leaves (Section 2.1);
- Translation of a subset of design principles into a family of two-dimensional abstract geometries, reflecting leaf “morphotypes” and some results of the shape analysis of oak leaves. Paper models were used as leaf analogs in “proof-of-concept” evaporation tests, to observe and compare the evaporative transfer of these leaf-inspired geometries (Section 2.3).

### 2.1. Biology Research: Shape Analysis of Oak Leaves

**Figure 1:**
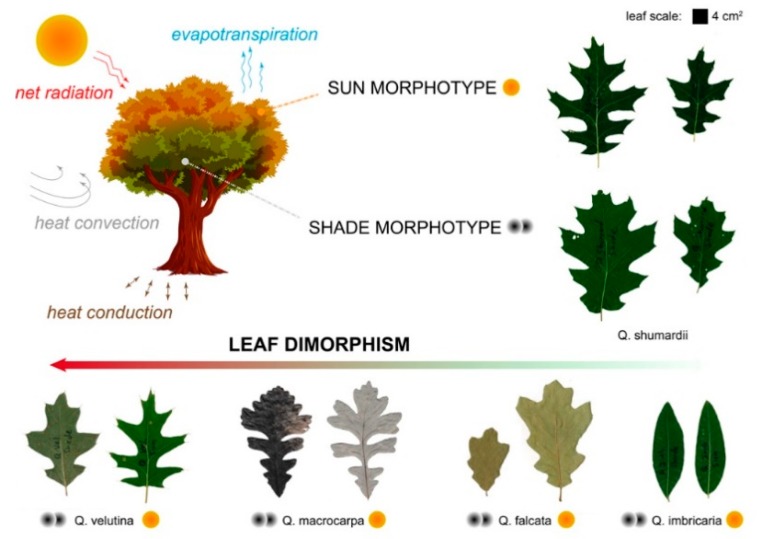
Plant thermal exchange budget and leaf heteromorphism, illustrated with sun and shade leaves from different oak species.

In this experiment, we studied heteromorphic leaves from different plants and taken from different parts of the canopy, for shape measurement and analysis with geometrical parameters. Leaf morphological plasticity can be found at the scale of the plant individual and is usually associated with microenvironmental differences within the canopy [29]. Sun–shade dimorphism is such an example, with leaves from the top and outer layer of the canopy, so-called sun leaves, differing from shade leaves, from the interior and lower levels (Figure 1). During summer 2017, fully expanded mature leaves were collected from mature trees of at least 11 identified oak species (*Quercus* genus), most native to North America: *Q. alba*, *Q. bicolor*, *Q. ellipsoidalis*, *Q. imbricaria*, *Q. falcata*, *Q. robur*, *Q. rubra*, *Q. nigra*, *Q. shumardii*, *Q. macrocarpa*, and *Q. velutina*. Leaves were collected at two different sites in Ohio (USA, East North Central region), the great majority from Holden Arboretum (Kirtland, OH) where some species were already identified. Otherwise, species were collected at the campus of the University of Akron (Akron, OH) and identified based on plant leaves, bark, and acorns. Both sites were the location of growth of the studied plants, thus all leaves were assumed to have developed in the same temperate and seasonal climate. Both sites are characterized by a humid continental climate of year-round precipitation, warm to hot humid summers, and cold snowy winters, i.e., Dfa/Dfb climate types according to the updated Köppen–Geiger classification [30]. Shade leaves were picked from the interior or bottom of the canopy and sun leaves from exposed or top branches, often with a reacher tool.

**Figure 2:**
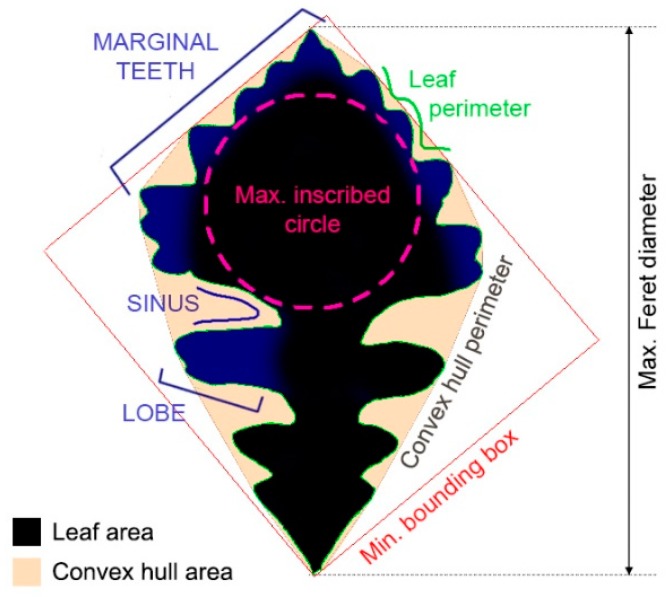
Leaf shape study based on qualitative features (e.g., leaf lobes, sinuses, marginal teeth) and quantitative parameters (e.g., perimeter, area, convex hull, inscribed circle).

Leaves were pressed flat and stored in a herbarium binder. Digital scans of the flattened leaves were analyzed with ImageJ (FIJI) software and ShapeFilter plugin, to compute geometrical regions of interest and two-dimensional shape parameters: Perimeter (P), area (A), aspect ratio, convex hull perimeter (H) and area (C), minimum bounding box, maximum inscribed circle, convexity, solidity, level of dissection index (LDI), roundness, and fractal dimension, for each leaf (Figure 2). Leaf convex hull was numerically determined as the smallest polygon enclosing the leaf contour. Convexity and solidity are defined, respectively, as the ratios of the convex hull perimeter to the leaf perimeter (H/P), and the leaf area to the convex hull area (A/C) [31]. The level of dissection index (LDI) is a size-independent morphology parameter commonly used in botany, defined as leaf perimeter^2^:surface area (P^2^/A), normalizing the margin extension to the leaf area. Roundness, calculated as 4πA/H^2^, is insensitive to leaf border irregularity (i.e., dissection) and tests the circularity of a shape’s overall spread, taking a maximum value of 1 for circles. The maximum inscribed circle diameter presumably gives the leaf effective width and characteristic dimension [32], an interpretation which is discussed in Section 4.3. Alternatively, a mathematical approach suggested in boundary layer theory applied to leaves was used for characterizing abstract geometries [33]:

(1) $$L_{\mathrm{eff}}=\sqrt[{n-1}]{\frac{\int_{0}^{w}\;Y^{n}\left(x\right)\mathrm{dx}}{\int_{0}^{w}\;Y\left(x\right)\mathrm{dx}}},$$

where L_eff_ is the effective dimension, W is the maximum shape width in the airflow x-axis direction, Y(x) is the variable distance from one edge to the opposite in the y-axis direction, and n is an empirically determined parameter depending on boundary layer laminarity and flow conditions (typically, *n* = 0.5 or *n* = 0.75 for a free convection regime) [33]. Otherwise, $\sqrt{A}$ was used for real leaves as characteristic leaf length, for tests on parameter size-dependence. While leaf aspect ratio was based on leaf maximum to minimum Feret diameter ratio, elongation e was calculated from the minimum bounding box side lengths (e = 1 − short side: long side). ShapeFilter estimation of the fractal dimension is based on a box-counting algorithm [31]. For each measured parameter, one-way analysis of variance tested if shape quantifiers significantly differentiated sun from shade leaves among all or within specific oak species (two-sample Student’s *t*-test assuming unequal variances, JMP statistics software). We used a significance level of α = 0.05 for all statistical tests. A total number of 206 leaves (96 sun, 110 shade) were analyzed.

### 2.2. Biomimetic Design Principles: Abstracted Leaf-Inspired Geometries

Hypothetical design principles were abstracted from leaf shapes and formulated (Section 3.2), based on the review of plant science literature, the experimental results with oak leaves, and our own previous exploratory work [34]. The present work looked closely at a subset of these design hypotheses, touching on aspects of lobation, toothed edges, and leaf blade elongation. A bio-inspired shape family of four two-dimensional geometries was tested and compared. Qualitative aspects of shape dissection were considered, namely the addition of geometrical features analogous to leaf protrusions (i.e., lobes and marginal teeth). Geometry protrusions were distributed as identical triangular units on circular outlines, with rotational symmetry, and in two size ranges to classify as lobes and marginal teeth, respectively, according to botany geometrical definitions [35]. Geometries were also fine-tuned quantitively, so that design sizes and proportions were of the same order of magnitude as in real leaves, and to yield disparate values for specific geometrical parameters (e.g., elongating the elliptical shape to increase its aspect ratio, tuning solidity by adjusting the size and number of marginal teeth). The bio-inspired geometries represent a first design iteration for analyzing structure–transfer relations, with substantial simplifications (e.g., rotational symmetry), which reduce the complexity of “elongate”, “lobed”, and “toothed” leaf morphotypes. The geometries were mathematically solved to have the same surface area, using different polygonal surface area formulas. To take a first look at scaling effects, they were scaled to two sizes (5- and 10-cm diameter), comparable to the studied broad leaves in their young and mature forms. Design was handled with Rhino 5, 3D CAD software tools. Grasshopper visual programming plug-in supported numerical computation of Equation (1), for a free convection regime (n = 0.75, but L_eff_ values little differed for n = 0.5).

### 2.3. Proof-of-Concept Evaporation Study with Bio-Inspired Paper Models

This experiment tested hypothetical bio-inspired principles to illustrate how leaf morphology may serve as a reference for exchanger design. The four abstracted geometries were applied to leaf analogs made of filter paper (Ahlstrom grade 631), a water-absorbing porous material of a known pore size (10 microns) and low thermal conductivity, analogous to evaporative leaf tissues [36]. The selected shapes were laser cut to high precision for two characteristic dimensions (i.e., circle diameter D = 5 cm, A = 20 cm^2^; D = 10 cm, A = 79 cm^2^), representative of smaller/younger and larger/older leaves. Models’ dry and wet weight were satisfactorily constant among the different replicas and shapes (within 5% error), thus local disparities in the filter paper’s porosity were considered negligible. Paper models were coupled with equally shaped thin plastic plates keeping the paper flat and restraining evaporation to one side (analogous to most terrestrial plant leaves, which have transpiring pores only on the lower surface). Models were wetted with purified water via water level rising for uniform wetting, as described in a previous work [34], then covered with sealing lids cut to size, and transferred to an analytical scale (Denver Instrument M-220, class I accuracy ±0.1 mg) inside an environmental chamber (Associated Environmental Systems, model BHD-408) for air temperature and relative humidity control, set to T_air_ = 35 °C and RH_air_ = 35%. Lids were kept for 1 or 2 h to hinder evaporation while models thermally adjusted to the chamber, and then removed to release the water vapor. Thin supports held the models in place horizontally oriented, elevated from the balance plate (8.5 cm high), for proper heat and mass exchange with environmental air.

Each shape was tested separately (N = 4) in the chamber for the same environmental conditions. The experimental setup tracked each model’s mass transfer by recording the evaporated water weight loss from the wet paper, though the scale’s RS232 serial port (1 measurement/10 s). Since the scale had its own enclosure, side walls were kept closed to protect the model from forced convection by the chamber’s air circulation (v_air_ < 0.1 m/s, measured with a hot-wire anemometer). The top was kept open, to ventilate water vapor and assure constant air temperature and humidity inside the enclosure, as maintained by the chamber. During evaporation recording, a humidity-temperature sensor inside the analytical scale accompanied slight temperature (±0.5 °C) and relative humidity variations (±7%) along chamber regulation cycles, consistent among all tests. A thermal camera (FLIR T430 series, 320 × 240 pixels) was also mounted inside the chamber to capture time-lapse thermograms (1/min) of the evaporative cooling effect for two of the shapes.

Model water content was not renewed, knowing that evaporation from water-saturated porous media under very low evaporative demand (gentle to still airflow) is typically characterized by an initial phase of a constant evaporation rate, unaffected by capillary phenomena and hydraulic shortfall [37]. Assuming diffusion is the primary mass transfer mechanism within the boundary layer developed by the surface, the evaporative flux, E (rate per unit area), may be estimated from the measured air temperature, t_air_, model average surface temperature, t_model_, relative humidity, H, and saturated vapor densities, n_s_, at the model surface (air assumed saturated) and in the free stream:

E = D_V_ [n_s_(t_model_) − H n_s_(t_air_)]/δ, (2)

where D_V_ is the diffusion coefficient of water vapor in air and δ is the boundary layer thickness. Assuming a laminar boundary layer, a first approximation may be given by δ ≈ 4.91$\sqrt{{\nu \;L}_{\mathrm{eff}}/v_{\mathrm{air}}}$ [33], with ν kinematic viscosity of air. Evaporation rates (weight loss rate) were taken as the slope values extracted from linear regression fits to the time series plot of models’ weight (Microsoft Excel 2013). Weight loss within the initial drying phase was confirmed to be linear with our setup (R^2^ > 0.999). Measured evaporation fluxes were compared via one-way ANOVA with JMP software.

## 3. Results

### 3.1. Biology Research Findings

#### 3.1.1. Leaf Role Models for Evaporative Thermal Design

Table 1 summarizes the studied plant species, and reported morphological patterns organized by type and extent of leaf shape variation. Through the literature review, we identified time- and space-related morphological trends having an impact on leaf exchange. For shape variation over time, some plants develop leaves of different shapes at different growth stages. The time scale for these variations can be seasonal, as in shape changes from winter to summer [38], or longer, over the lifespan of a plant individual, such as in young shrubs carrying leaves that are differently shaped from the mature trees—an occurrence designated by botanists as heteroblasty [27]. Heterophilly is the more general term for any instance of a plant carrying leaves with distinct morphologies and functions, and encompasses space-sensitive changes. Regarding morphological variation over space, the study of global leaf patterns and climatic adaptation is an active research field, with solid findings about latitude-sensitive leaf shape features, namely leaf elongation in warmer climates for the *Viburnum* clade [39] and development of marginal teeth in colder climates for woody flowering plants (dicots) [40]. At the scale of the individual plant, shape diversity may be found within the canopy. Sun–shade leaf dimorphism is the most documented case since it affects numerous plant species [41]: Leaves on the top and outer shell of the canopy (called sun leaves) typically become more lobed or dissected than internal and lower level “shade” leaves, which are less exposed to the elements [42]. Shape trends may be found even at the scale of a single leaf, as in the premature development of spongy mesophyll tissue with an evaporative role at the leaf margin [43], or preferential lobation on the leaf’s basal side [44].

These leaf shape trends constitute a promising new biological solution space for bio-inspired thermal devices, from which leaf-inspired design principles will be later abstracted (Section 3.2). From a botanical standpoint, broad-leaved flowering plants (Eudicot Angiosperms) from temperate to tropical climates were considered the most pertinent biological role models, given their exposure to seasonal environments, diversity of leaf shapes in general, and multiple studies documenting morphology relations to plant thermal function. Further arguments for this selection will be discussed (Section 4.2), touching on the functional significance of leaf shape for the identified case studies and the suitability of plant leaves from temperate to tropical climates to inspire thermal exchanger design.

#### 3.1.2. Quantifying Oak Leaf Dimorphism

The objective of this experiment was to quantify shape differences between sun and shade leaves across multiple North American species of the *Quercus* genus, whose leaf morphology and plasticity has been previously addressed in the literature and hypothesized to serve a thermal purpose [42,50]. Table 2 identifies geometrical parameters that characterize sun–shade dimorphism across different oak species, despite species-specific leaf shape features Shape parameter values for all leaves and averages for each oak species are featured in Tables S1 and S2 of the Supplementary Material.

Perimeter (*p* < 0.0001), LDI (*p* < 0.0001), convexity (*p* = 0.0002), and roundness (*p* = 0.0012) most significantly differentiated sun from shade leaves, among all oak species: Sun leaves had longer margins in both absolute and relative terms and were less circular. Other shape parameters differentiated sun leaves with significance: Lower solidity (*p* = 0.006), longer convex hull perimeter (*p* = 0.007), longer maximum Feret diameter (*p* = 0.009), larger convex hull area (*p* = 0.011), larger minimum bounding box (*p* = 0.008 for long side, *p* = 0.016 for short side), and longer minimum Feret diameter (*p* = 0.017). Some shape parameters did not significantly characterize the dimorphism: Area-equivalent circle diameter (*p* = 0.063), area/perimeter ratio (*p* = 0.09), leaf surface area (*p* = 0.09), maximum inscribed circle diameter (*p* = 0.18), fractal dimension (*p* = 0.2), elongation (*p* = 0.5), and aspect ratio (*p* = 0.5).

Since sun leaves were not significantly larger, their longer perimeter was due to shape rather than size, as given by a higher LDI, which normalizes perimeter to area. Besides, LDI (R^2^ = 0.0003), convexity (R^2^ < 0.006), and solidity (R^2^ < 0.07) did not significantly correlate with the characteristic leaf length ($\sqrt{A}$), suggesting that oak leaf shape is independent of size. Sun leaves’ significantly higher LDI and lower convexity reflect the extension of the border perimeter within a limited area, which is possible via shape dissection with more numerous and/or proportionally larger lobes (*Q. falcata*) or marginal teeth (e.g., *Q. macrocarpa* crown, *Q. bicolor*). These first results support the general observation that oak sun leaves are more deeply lobed and point to the possibly important role of the leaf border in exchange performance. They also show that simple parameters can quantify sun–shade dimorphism, and such parametrization may guide the design of dissipative exchangers inspired by sun leaves.

We also tested the same shape parameters within *Q. alba*, *Q. macrocarpa*, *Q. ellipsoidalis*, and *Q. bicolor* samples, to consider species-specific leaf morphological traits. This selection targeted particular design features: Highly variable degree of dissection in *Q. alba*, combination of lobes and marginal teeth in *Q. macrocarpa*, a relatively low number of widely spaced lobes in *Q. ellipsoidalis*, and development of marginal teeth only in *Q. bicolor*. Table 3 presents sun vs. shade results for dissection-sensitive quantifiers of LDI and solidity.

**Figure 3:**
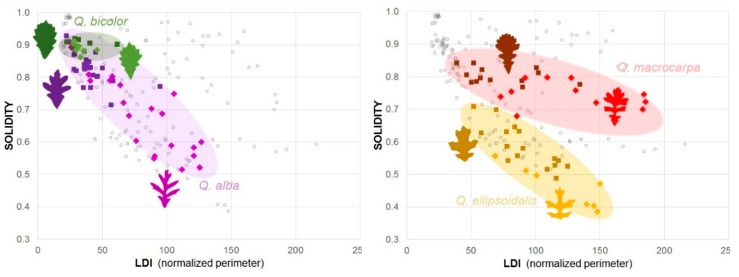
Sensitivity of shape parameters to sun leaf dissection in *Q. bicolor* (left, green dots), *Q. alba* (left, purple dots), *Q. macrocarpa* (right, red dots), and *Q. ellipsoidalis* (right, yellow dots). The species’ datapoints are grouped and emphasized by merely qualitative colored sets. Hollow dots represent the other oak species’ leaf datapoints from the collected sample.

Except for *Q. bicolor* (N_sun_ = 10, N_shade_ = 10), whose sun–shade subtle differences were statistically insignificant for the used parameters, trends for individual oak species were similar to the cross-species sample, with sun leaves significantly more dissected. Moreover, aspect ratio (*p* = 0.007) and elongation (*p* = 0.03) were significantly lower for *Q. ellipsoidalis* sun leaves, whose relatively longer lobes reduced the ellipticity of the overall leaf shape. Solidity was an effective parameter to differentiate lobation from toothiness. Both cases extend the leaf border (perimeter), but relatively small marginal teeth do not expand the leaf convex hull area as much as a few, relatively large lobes do, even when both yield similar LDI values. For instance, *Q. macrocarpa* and *Q. bicolor* toothed morphologies were identified as more “solid” than more dramatically lobed ones (*Q. ellipsoidalis*). The tendency of sun leaves to morph towards higher LDI and lower solidity is represented in Figure 3. Pairwise tests gave the following negative correlations between solidity and LDI (sun and shade leaves): *Q. alba* r = −0.90 (*p* < 0.0001); *Q. ellipsoidalis* r = −0.86 (*p* < 0.0001); *Q. macrocarpa* r = −0.58 (*p* = 0.0028); and *Q. bicolor* r = −0.15 (*p* = 0.53). These correlations link leaf shape dissection in oak species to border extension.

Concerning other oak species, the highest LDI values in the studied sample were found for *Q. rubra*, featuring fractal-like marginal dissection with simple to spinose hierarchical teeth, an interesting concept to explore in bio-inspired thermal exchangers. Some oak species were not as dimorphic and even lacked any form of dissection (e.g., *Q. imbricaria*), which illustrates the phenotypic variation within the genus. *Q. nigra* results were opposite to the mainstream, with solidity differences not significant (*p* = 0.6), and shade leaves with higher LDI (*p* = 0.002) and higher elongation parameters (i.e., elongation *p* = 0.02, aspect ratio *p* = 0.04). This exemplifies leaf elongation as a third strategy for border extension (e.g., ellipses have greater relative perimeters than circles).

### 3.2. Leaf-Inspired Design Principles

#### 3.2.1. Geometrical Abstraction from Leaf Role Models

Within the solution space of leaf morphology tuned for plant thermoregulation, we were able to discern shape features of interest, summarized in Table 4.

Despite the great diversity of leaf shapes gathered along this investigation, the goal was to distill common morphotraits for dissipation, not associated with any plant species in particular. Table 4 takes a first step toward hypothesizing the relation between transfer enhancement and specific design features that were characteristic of the identified leaf role models: Blade elongation or fragmentation, the relative size and shape of protrusions (or of the space between them), and hierarchical design combinations. These design hypotheses are either unaddressed or have been explicitly stated in the literature but not tested (i.e., *Viburnum* and oak references [39,48]). Hypotheses were organized according to the observed or conjectured thermal modes for leaf exchange (i.e., sensible and/or latent heat and/or mass transfer regime, from simple convective cooling to evaporation to combined phenomena as in evaporative cooling). Future work on validating these hypotheses, identifying potential thermal applications and developing a physical understanding of the design–transfer relations, will facilitate biomimetic abstraction and translation of these findings.

#### 3.2.2. Leaf-Inspired Morphotypical Geometries

Table 5 introduces four geometries resulting from bio-inspired abstraction and envisioning thermal design based on leaf shape. Shapes E (for “elongate”), L (for “lobed”), and T (for “toothed”) provide a first step in addressing *Viburnum* and *Acer* design hypotheses from Table 4. To assess the impact of teeth proportions, the “teeth” of shape L were enlarged to the point of fitting the biological definition of “lobe”. This also emphasizes the difference between densely toothed margins (T) and large-scale lobation (L), when comparing shapes L and T. The diversity in oak leaf morphology called for such distinction, with both strategies—development of few, relatively large, or many small protrusions—found in sun leaves. With generic representatives of entire, elongate, lobed, and toothed leaves, we hope to give a broad scope to what may constitute a dissipative morphotype. With geometrically simplified versions of each morphotype, we also hope to facilitate the uncovering of new shape–transfer relations in leaves.

The candidate shapes were tested for their ability to dissipate water vapor. In light of the parameters used to characterize oak sun–shade dimorphism, the selected shape quantifiers were considered. The morphotypes were geometrically tuned to have distinct LDI, effective width (maximum inscribed circle diameter), convexity, and solidity. Shape E addressing leaf blade elongation was the only geometry with a non-unitary aspect ratio (ellipse aspect ratio = 2) and the smallest effective width and dimension L_eff_ (Equation (1)), and thus was expected to result in higher dissipation rates. However, the evaporation results presented in the following section reveal that none of these shape parameters completely dictate dissipative performance, demonstrating how difficult it is to theorize geometry–transfer relations, especially for mass transfer under low airflow conditions.

### 3.3. Proof-Of-Concept Evaporation Tests

The evaporative performance of above-described leaf-inspired geometries was inspected with filter paper models. Given the size of the models and experimental conditions with airflows below 0.1 m/s, geometries were tested in a transfer regime between free mass convection-dominance (i.e., resulting from humidity gradients only) and mixed convection (i.e., combined free and forced convection). The lowest limit to the Richardson number discards forced convection-dominance (i.e., Gr/Re^2^ = 1.3), a less thermally challenging regime for real leaves, as will be discussed in Section 4.2.4.

**Figure 4:**
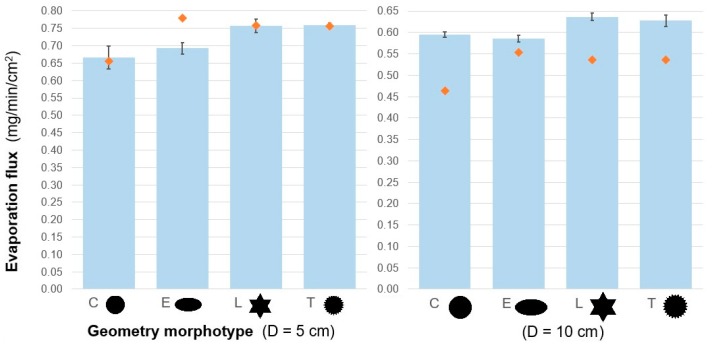
Mean evaporation flux for filter paper models with leaf-inspired morphotypes, for two characteristic dimensions (control circle D = 5 and 10 cm). Error bars represent ±1 SD. Orange markers represent theoretical estimates based on L_eff_ (Equation (2)), for t_model_ = 28 °C and v_air_ = 0.015 m/s.

Figure 4 and Table 6 feature evaporation flux values and ANOVA results assessing shape-related differences. Shape-related differences in the evaporation rate were significant at either characteristic dimensions (*p* < 0.0001). Both toothed and lobed models evaporated significantly faster than the control and elongated models, an enhancement which was more pronounced, up to 14%, for the smaller characteristic dimension (control D = 5 cm). Evaporation flux estimates in Figure 4 (orange markers), presupposing L_eff_ to relate to boundary layer thickness through Equation (2), were overall underestimated for the larger models (D = 10 cm) and overestimated for shape E.

**Figure 5:**
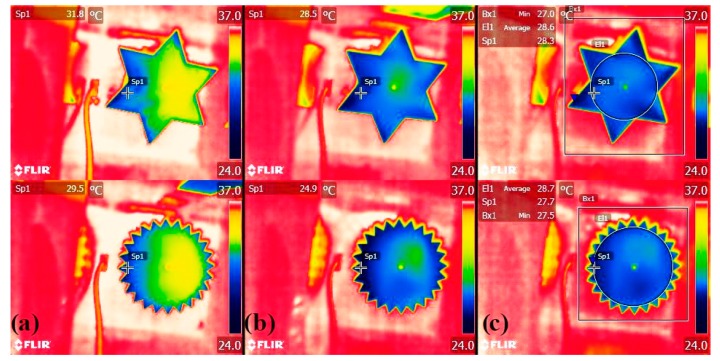
Thermal time-lapse of evaporating models of lobed and toothed shapes (characteristic dimension 10 cm): (**a**) Shortly after cap removal (evaporation release); (**b**) 1 min after; and (**c**) 20 min after.

The cooling effect of evaporation was tracked for L and T large models. Thermograms (Figure 5a) show the effect taking place right after cap removal, with model surface temperatures initially distributed in an asymmetric manner because of heterogenous airflow exposure. Surface temperatures, lowered from phase change energy loss, became more uniformly distributed only 1 min after uncovering (Figure 5b), with lowest temperatures concentrated near the models’ border. More dramatic local cooling was located on the marginal teeth of model T (Figure 5b), up to 10 °C below air and 2 °C below the maximum local cooling observed for model L.

This highlights the potential for shape-induced differences in cooling dynamics and the advantage of finely toothed edges for thermal applications where maximum local cooling or mass transfer is required. Our results also contrast with non-transpiring models where toothed edges did not lead to observable differences in convective cooling [48]. However, it seemed both models maintained similar average temperatures, consistently 7 °C below air, even though changes in average surface temperature over time were not tracked. The impact of the teeth relative size, from merely local enhancements to effective gains in the overall cooling and dissipation rate, is an interesting design question to be further investigated [53]. Experimental variability in evaporation rates was also particularly low for the smaller T model (D = 5 cm), pointing to a stable mode of transfer—another aspect to be tested, as transfer in dissected geometries is expected to become less dependent of orientation [48].

## 4. Discussion

### 4.1. Mass Transfer Enhanced by Geometry Dissection

Leaf-inspired geometries, namely dissected surfaces, were shown to have a positive impact in mass transfer and evaporative cooling. Dissection as a general design principle, here based on observations and hypotheses known to plant science, is also found in the literature of passive techniques for enhancing convective heat transfer (e.g., structured and extended interfaces “by shaping or interrupting the surfaces” [54]). Nonetheless, shape enhancements in the heat transfer literature are usually proposed under the principle of increasing surface area, while this and previous work [34] indicate that shape only also affects transfer, aside from surface area or even border length (i.e., perimeter). Since evaporation differences between L and T morphotypes were insignificant, despite shape T’s larger LDI, similar transfer enhancement may be achieved via different geometrical strategies. This also indicates that maximizing border length (e.g., via finely toothed edges) is unnecessary, an important learning to consider for hierarchical structures. Current research addresses similar cases of limited conductive or convective transfer in fractal geometries [55], constructal designs [56], and branched networks [57], where proceeding with further hierarchization of a design does not always enhance transfer. A recent review emphasized the inconclusive status of thermal design optimization based on hierarchization principles, the predominance of analytical or numerical approaches, the need for more experiments, and the lack of research on liquid-to-gas heat exchangers [58]. Therefore, proceeding with the proposed biomimetic research seems opportune.

All smaller versions of our paper models (D = 5 cm) had slightly higher evaporative fluxes (rate per unit area), expected from their thinner boundary layers and easier transfer with the environment [33]. Shape-related differences were less significant for the larger models (D = 10 cm), with dissection increasing evaporation rates by 6% to 7% only. That dissection may not always have the same thinning effect on the boundary layer calls for further research on up-scaling leaf-inspired exchanger structures and the definition of consistently dissipative geometries. From a botanical perspective, changes in leaf size arise as a possible factor for the drop in marginal teeth transpiration past the growing season [47]. We also hypothesize that transfer in very large leaves, namely those of tropical plants, is rather enhanced by more extreme forms of dissection effectively downsizing the overall leaf scale, such as pseudo-compoundness (e.g., *Trevesia palmata* case study), than lobation and marginal teeth. If true, this hypothesis would add another argument explaining the global pattern of shape entirety found for tropical leaves [39].

The mechanisms and fluid dynamics explaining the interplay between dissected surfaces and their resulting boundary layers, in low airflow conditions, remain to be described. Our experiment suggests that border extension in flat interfaces, even though important, is not the only mode for transfer enhancement, since large-scale dissection can be equally effective. Transfer may be facilitated by different forms of flow phenomena, related to the following: (1) Large-scale dissection, inducing three-dimensional modulation and overall thinning of the boundary layer; and (2) small-scale or marginal dissection, affecting turbulence—as seen in other applications with toothed edges [59]. The setup used here was limited to simple mass transfer, for one set of environmental conditions only, calling for future experiments with radiative loads and coupled heat and mass convection, testing more factors for thermal decoupling from the environment. This would clarify the set of environmental conditions and thermal applications best suited for biomimetic translation of leaf functional morphology. Further studies with leaf analogs would also support the science of leaf-air interfaces, which lacks empirical estimates of the boundary layer resistance to transfer at low windspeeds [33].

### 4.2. Biological and Biomimetic Significance

#### 4.2.1. Heterophyllous Leaf Dissection

Most plant role models identified in this biomimetic effort are heterophyllous, i.e., carry leaves of distinct morphologies. Heterophilly can be a mere byproduct of changes in environmental conditions during growth but has been shown to have adaptive significance in certain cases, by improving leaf function for the local microenvironment. A well-documented case of functional heterophilly is aquatic plant species whose submerged leaves are drastically more dissected than those above water to overcome the aquatic environment’s lower diffusivity and facilitate gas exchange [60,61]. For land plants, dissected shapes are also hypothesized to allow faster exchange and dissipation, which may explain morphological patterns, such as lobation and development of marginal teeth. In general, dissected surfaces hold thinner boundary layers since the layer of unstirred air adjacent to the surface is unable to develop further within the surface, located an adequate distance away from the surface edges, which are better ventilated by default [33]. This would explain the documented *Viola septemloba* and mulberry leaf temperatures, which were significantly lower for dissected and many-lobed leaves in a sun situation (down to 6 and 3 °C, respectively [39,49]). The boundary layer is expected to insulate the leaf from the environment, reducing heat and mass exchange and retaining a microclimate over the leaf, which some insects may even rely on for egg laying [62]. However, arguments from boundary layer theory have usually been transferred directly to botanical discussion on leaves without empirical confirmation and therefore need further research.

#### 4.2.2. Sun–Shade Leaf Dimorphism

Leaf sun–shade morphotypes are defended to be an adaptive response to different environmental conditions within the canopy. Light is a strong selective pressure on leaf function and may explain the dimorphism: Sun leaves would allow better light penetration through the canopy, to reach shade leaves [29,42]. The *Trevesia Palmata* case study from our literature review refers to a tropical/sub-tropical plant whose sun leaves are more dissected, even becoming pseudo-compound in mature plants [51]. Whether such shape plasticity originates from light adaptation is unknown. The heat tolerance of transpiring shade leaves in tropical plants may be only marginally lower than in sun leaves [63]. However, shade leaves are adapted to use diffuse light [42], and the relative importance of leaf shape gradients, among many other factors affecting canopy light penetration, has not been assessed in the field yet.

An alternative—or likely complementary—evolutionary explanation for the dimorphism is related to leaf thermal management, as in avoiding above ideal or even lethal temperatures from being more exposed to solar radiation and drastic changes in temperature [50]. Given that sun leaves are typically more lobed or dissected [41], the same theory for geometry-mediated thinning of the boundary layer equally applies. Other morphological aspects of sun leaves besides blade shape provide further arguments for their thermal function. Sun leaves also have a higher number of pores for transpiration (stomata) [29] and are usually smaller and thicker [49]. The additional thickness results from further tissue development for light management and gas exchange [29], and may increase sun leaves’ heat capacitance, slowing down heat gains from sudden solar exposure (i.e., light flecks) [64].

The structural differences of sun leaves may facilitate mass convection of water vapor and evaporative cooling (latent heat loss), or simple convective cooling via heat exchange only (sensible heat loss). As seen with the *Viola septemloba* and *Geranium sanguineum* case studies, sun leaf-like morphotypes are also found in plants challenged by water scarcity, either during summer or growing in drier environments [45]. Dissected leaves may be more tolerant to drought, as decreased leaf temperatures from sensible heat loss reduce evapotranspiration and help the plant conserve water. For the specific case of oak morphology, non-transpiring sun leaf-shaped models were empirically shown to better dissipate sensible heat [48]. Conversely, non-transpiring shade leaves can suffer heat damage under a sunfleck on a hot summer day within only one minute [65]. Sun leaves’ higher capacity for evapotranspiration has been observed in *Q. rubra* specifically, reaching significantly lower temperatures of down to 4 °C compared to shade leaves on the top of the canopy, on a sunny midsummer day [50]. Such a thermal difference was reduced with both leaf types positioned at the bottom of the canopy, under much lower levels of irradiance. This, and the fact that sun and shade leaves were equally shaped at the time of bud break, led to the conclusion that morphological adaptation is governed by microclimatic differences in evaporative demand. However, how plants with heteromorphic leaves in water-limited contexts balance physiological (e.g., metabolic regulation of stomatal aperture) against structural (e.g., stomatal and/or leaf morphology) strategies to reduce evapotranspiration remains unclear. Nonetheless, sun leaves are a promising bio-inspiration for structure-based convective and evaporative cooling. A future interesting biomimetic research theme are evaporative geometries able to decouple heat from mass transfer, especially for passive exchangers where evaporation cannot be suspended whenever unnecessary (e.g., water-saving applications in building facades, when wind alone provides enough convective cooling).

#### 4.2.3. Marginal Teeth

From biomechanical constraints to the consequences of bud packing, many hypotheses to explain the development of toothier leaves in plants from colder climates are still debated [66]. This global pattern is a well-established climate–leaf shape relation found among woody species, even used in paleoclimatic reconstruction based on leaf fossils [35]. The early-season growth hypothesis highlights leaf mass exchange: Toothed leaves enhance marginal evapotranspiration and carbon uptake rates, boosting photosynthesis during the short time window for growth in cold but water-available climates [67]. Such a hypothesis is supported by local measurements of transpiration, which were found to be considerably higher at the toothed margins than at the leaf centers in a study on 60 plant species (including maple, oak, and one *Viburnum* species) [47]. Fast-growing plants may be good candidates for studying fast leaf transpiration and mass dissipation enhancement in thermal exchangers. However, this enhancement is significantly lost once the leaves mature. This calls for further research on the relative contributions of: (1) Prematurely developed evaporative tissues within teeth and margins in juvenile leaves [43]; (2) metabolic and/or anatomical changes (e.g., hydathode occlusion [67]); and (3) geometrical changes, such as the effect of leaf size and allometry (e.g., proportionally larger teeth in young leaves) on boundary layer thinning. Leaf margin design is often overlooked in the description of leaf morphology and its potentially non-negligible effect in transfer is discarded [33]. Thus, toothed edges as a design feature for dissipation were considered in this study.

#### 4.2.4. Leaves from Temperate to Tropical Climates as Exchangers

The selection of broad-leaved plants from temperate to tropical climates as a source of bio-inspiration for thermal exchangers was not arbitrary. While temperate climates are not continuously challenging, their impact on plant function may be examined at different time scales to reveal considerable variability along seasonal and day–night cycles, regularly resulting in episodes of critical thermal pressure [48]. In contrast, the most thermally challenging conditions plants are known to endure are often coupled with extreme water scarcity, such as in deserts and high altitudes. Water conservation then becomes the most pressing design constraint, leading to the particular strategies and morphotraits found in xerophytic plants: Pubescence, limited diurnal evapotranspiration, and modified leaves with lower surface area-to-volume ratio, such as succulents or conifer needles [68]. While xeromorphic plant design may provide insight into insulative, radiative, thermal lagging, and water conservation strategies [69], it is distant from an interpretation of leaves as evaporative exchangers. For phase change and fluid-assisted thermal systems, convective exchange by leaves in water-available environments are a likely better biomimetic fit to inform structure-based fast dissipation and evaporative cooling.

Furthermore, non-xeromorphic plant leaves still face environmental risks. On a hot day, they may experience several heat load peaks because of directly hitting sunflecks and low wind conditions. Even if still air is temporary and rare outdoors, it is exactly those less frequent but episodically extreme conditions that are expected to pose a real threat to leaf tissues, according to energy balance calculations [48]. At night, wind combined with very low air temperatures may cause desiccated leaf margins known as “wind burn” damage. A cloudless night sky is also a heat sink, reducing atmospheric absorption of net radiation and possibly freezing leaves, which often reach below air temperatures via radiative cooling. Recent studies of leaf size global trends actually point to a stronger correlation between size and nighttime cold constraints—rather than daytime heat—in wet sites [70]. Therefore, worst-case scenarios represent a significant selective pressure for thermoregulatory adaptation, as do average conditions [33,71]. Regarding the *Viburnum* clade, leaf roundness and the number of marginal teeth were more strongly related to the mean temperature of the coldest quarter than with annual averages [39]. This provides a first hint into climatic criteria for the search for leaf role models with thermally significant morphology. However, the question of whether sun–shade dimorphism is related to the avoidance of local leaf damage (e.g., protection from extreme overheating or radiative cooling) or to optimization of overall canopy photosynthetic gains [42] (e.g., light penetration hypothesis) requires further research.

#### 4.2.5. The Multifactorial and Multifunctional Realm of Leaf Morphology

To conclude on the functional significance of leaf shape, its evolutionary dimension drags a phylogenetic history, which may or may not be adaptive for the present environment, and must be taken into account when seeking bio-inspired design principles [27]. Leaf plasticity even so markedly influenced by environmental conditions and defended to serve evolutionary purpose [72], has genetic and developmental constraints [73]. Besides the uncertainty about leaf shape fitness gains, large-scale studies on climatic patterns emphasize the complex relations between morphology and multifunctionality, likely leading to many equally viable strategies for a set of environmental conditions [70]. Nonetheless, we see here an opportunity for design learning, in such a morphologically diverse group of plants as are broad-leaf angiosperms.

Morphological aspects beyond shape also impact heat and mass exchange and go beyond the frame of this research. Namely, topography and pubescence [74,75], hydathodes and wax plugs [67], orientation, photo/thermonasty (e.g., leaf folding, curling), fluttering, and reconfiguration in wind [48]. Leaves are not static organs, with petiole design and/or laminas of extended apex (e.g., acuminate to aristate shapes) possibly boosting fluttering in low wind [76] and circumventing boundary layer effects. Special attention must also be given to the leaf’s surface pores regulating water vapor fluxes, i.e., stomatal design, which greatly affect the plant transpiring response to the environment. Stomata–boundary layer interactions give leaf thermoregulation a non-linear dynamic, switching between dominant evaporative cooling to other thermal modes decoupled from mass transfer [77]. For leaf shape per se, water relations, more than thermoregulation, and cross-species studies have been identified as promising research directions for interdisciplinary investigation [27]. Both were considered in this manuscript, through the study of leaf morphology from different oak species and of water loss from shaped models (Section 3.1.2 and Section 3.3).

### 4.3. Applicability of Shape Parameters

#### 4.3.1. Sun–Shade Leaf Morphology

Some results from our shape analysis of oak leaves contrast with other studies. First, sun–shade aspect ratio differences were not significant: Elongation patterns found in *Viburnum* [39], where leaves become relatively narrower in hotter environments, do not extend to oak sun leaves. Second, we found no significant sun–shade differences for the diameter of the largest circle fitting within the leaf margin (i.e., maximum inscribed circle diameter), as shape and size variability within either leaf morphotypes was overridden, even within individual oak species. From the perspective of transfer science, such a parameter may be used to portray the leaf effective width (i.e., characteristic dimension), as the maximum fraction of the leaf surface equidistantly away from the border, available for boundary layer development [32]. The fact that dissection, not leaf effective width, was significantly different seems to oppose a recent study on the *Proteaceae* family (68 species) observing that the latter, rather than the former, predicts leaf thermal coupling with the environment (i.e., reduced insulation by the boundary layer) [32]. However, the study did not consider subtle but important transfer regimes, such as gentle airflow (between 0.2 and 0.5 m/s)—a forced convection-dominant regime for small leaves—from still airflow (below 0.2 m/s) combined with high solar load, a more potentially hazardous regime for most leaves (i.e., mixed free and forced convection) [48]. Such a distinction matters because the expectation of smaller effective width in leaves avoiding thermal stress relies on the direct relation between boundary layer thickness and the distance across a surface from the windward edge, only valid in forced convection-dominant regimes [33]. However, when transpiration is high and external airflows are very low, cross flow caused by surface evaporation may not be negligible anymore, introducing turbulence and mass transport cooling phenomena into the boundary layer [78]. In such cases, the details of 3D modulation of the boundary layer by surface geometry remain to be described. Therefore, the relative importance of effective width and shape per se in leaf thermoregulation needs further experimental investigation. Our experiments with paper analogs in a mixed convection regime show that effective width does not predict mass transfer performance (Section 3.3), an important learning for leaf-inspired evaporating structures.

To close on the further botanical work needed, a larger sample including more toothed leaf specimens would have better clarified the unknown interplay between sun–shade dimorphism and marginal dissection associated with cold climates. Further, there are limitations to single-parameter metrics, the shape analysis approach taken in this study. The specific descriptors used for detecting leaf dissection (i.e., LDI, solidity) did not capture *Q. bicolor* and *Q. nigra* subtle differences in marginal irregularity and toothiness, highlighting the possible advantage of shape-preserving image analysis techniques [79] and the usefulness of multi-scale morphometrics. Shape analysis so-called “landmarks” could be used for contour inflections, sinuses, or protrusion tips to assess hierarchical levels of border waviness [80]. Slight skewing in some leaves also distorted dissection results, because of sections of concave curvature along the leaf margin, directly affecting convex hull estimation. Principal component analysis (PCA) could have been used to combine multiple descriptors and study border curvature [39], such as in *Q. imbricaria*, whose sun leaves were found to be less elliptic and more ovate. Nevertheless, this study did not intend to exhaust the details of oak leaf morphology but to provide a first analysis of sun leaves’ dissection, not quantified before, and to orient the biomimetic abstraction and testing of design principles.

#### 4.3.2. Shape Parameters Relation to Evaporative Performance

Shape-driven transfer differences are explained by boundary layer theory, which takes into consideration how the geometry of an interface modulates surrounding flows and affects convective exchange. Geometries that thin the boundary layer are expected to enhance convection. The thinner the boundary layer, the less the resistance to transfer, and the faster the evaporation from a transpiring surface. However, capturing shape in boundary layer physics is difficult, leading to uncertainty when defining a geometry’s characteristic dimension [33]. Oversimplification may result in a complete loss of geometrical information beyond scale (e.g., √A, taking the exact same value for our paper model morphotypes of an equal surface area). An “effective dimension”, L_eff_, defined by a weighted mean (Equation (1)) is proposed in botany literature [33,81] as a geometrical proxy for boundary layer thickness, which accounts for shape characteristics beyond scale.

However, the effective dimension, L_eff_, was not a good predictor of evaporative performance for the tested paper models, as it was markedly overestimated for the elongated shape (E) and overall underestimated for the larger models (D = 10 cm). The computation of this theoretical parameter relies on the assumption that toothed edges do not interrupt flow–surface contact—an unlikely scenario for geometries with large-scale teeth—and is a good approximation only in either forced or free convection-dominant regimes, not mixed [81]. In our experiment, the relation between boundary layer thickness and L_eff_ was likely compromised by the mixed flow conditions, especially for shape E, which was more resistant to transfer than estimated. For the larger models, whose evaporative fluxes were overall underestimated, results might also have diverged due to unaccounted evaporation-driven turbulence introduced in the boundary layer, causing it to be thinner than predicted. Nonetheless, because shapes L and T had similar L_eff_ and evaporation performances, L_eff_ is possibly more accurate than the inscribed circle diameter approach [32] as a geometrical predictor of boundary layer thickness over complex leaf geometries (e.g., lobed leaves). For simpler, non-dissected shapes, and flow regimes more largely governed by forced convection, the minimum Feret diameter gives an edge-to-edge length dimension that is easier to measure than the inscribed circle diameter and likely suffices in predicting transfer performance. In field research, leaf width strongly correlated with leaf–air temperature differences (indicative of boundary layer thickness) within a wind speed range up to 1 m/s [82].

Finally, evaporation performance could not be inferred from the quantifiers that strongly differentiated sun and shade oak leaves (i.e., related to border length, shape parameters from Table 5). However, evaporation rate differences were too subtle for adequately testing dissection-sensitive parameters, such as convexity and solidity. Experiments with iterations and exaggerated versions of lobed and toothed morphotypes would be elucidative, before discarding the use of size-independent and easy-to-compute parameters in the design of leaf-inspired thermal exchangers.

We propose the translation of the biomimetic findings into thermal exchangers with finned designs as a starting point—typically, arrays of parallel flat plates. Non-rectangular plates with dissected geometries and/or non-smooth edges are to be explored: From simple concave polygons and serrated borders, to more space-filling structures, such as fringes and tortuous ramifications. Possible drawbacks of surface over-fragmentation might be avoided by taking leaf shapes with a high surface area to solidity ratios as role models. From our sample, *Q. falcata*, *Q. shumardii*, and *Q. velutina* are such examples. That both large- and small-scale teeth can deliver similar transfer enhancements is a useful learning for the design of dissected plates. Having options in thermal design is valuable because of the unique advantages of each geometry (e.g., compactness of shape T vs. potentially easier fabrication of shape L, given its relatively large shape elements). Future work and prototyping will be carried out to test multi-scale combinations of lobed and toothed design features and their three-dimensional translation into evaporative structures of porous media.

## 5. Conclusions

Thermal design is a real-world challenge with repercussions in energy management and concerning multiple technical applications. Most structures in thermal products originate from reiteration of long-standing engineering know-how, as more complex geometries are too difficult to simulate, produce, and explore. Because living systems endure thermal pressures too, biomimetics may offer an alternative path to design innovation. This interdisciplinary investigation shifts the focus to plant leaves’ thermal and water relations, governed by life-sustaining exchange budgets and continuous exposure to the elements. The interpretation of leaves as structural exchangers led this investigation to identify broad-leaf plant case studies promising to inform heat and mass transfer enhanced by shape. Even though a great diversity of leaf forms exists, and their adaptive significance is still disputed, certain instances of shape plasticity and morphology–environment patterns illustrate the thermal and evaporative function of leaf design. A trend for border extension and dissection in leaves expected to deal with higher thermal stress and evaporative demand was found across multiple species of oak. The identified shape patterns guided the formulation of design hypotheses and definition of abstract geometries emphasizing dissection aspects of leaf morphology. Evaporation tests with paper models of such “morphotypes” reinforced the conclusion that shape dissection, rather than border extension, enhances mass transfer in low airflow conditions. These research results lay the groundwork for leaf-inspired dissipation and introduce a novel source of biomimetic design learning, valuable for structurally improved water-assisted thermal exchangers.

## Figures and Tables

**Table 1:** Literature review on leaf exchange and morphology: Selected role models.

Leaf Shape Variation	Plant Case Studies, Reported Observations	References
**Temporal variation:** SEASONAL • summer vs. winter heterophylly	**Southern coastal violet (*Viola septemloba*)**: Developed more lobed leaves in summer, which maintained lower leaf temperatures	[38]
**Spatial variation:** GLOBAL • geographical trends • plant local adaptation	***Geranium sanguineum***: Leaves develop more elongated lobes in drier, continental habitats.	[45]
	***Viburnum* (*Adoxaceae*) clade**: Leaves in warmer climates are more elongated and entire, overall.	[39]
LEAF • developing leaves • margin transpiration	**Maple (*Acer* genus)**: Greater transpiration at the margins of young leaves; leaves grown in colder environments become more dissected, develop more numerous and larger marginal teeth.	[46,47]
	**Elm (*Ulmus* genus)**: Leaf tissues with evaporative role develop prematurely at the margins of young leaves.	[43]
PLANT CANOPY • sun vs. shade leaf dimorphism	**Oak (*Quercus* genus)**: Sun leaves have deeper lobes and greater transpiring capacity; transpiring sun leaves reach colder temperatures than shade leaves; sun leaf models in low wind convect heat better, more independently of orientation thanks to sinuses.	[40,48,49,50]
**Combined variation** **temporal and spatial:** PLANT LIFETIME • heteroblasty • young vs. mature plants	**Snowflake Aralia (*Trevesia palmata*)**: Sun leaves are more dissected; young plants have palmately lobed leaves, mature plants have pseudo-compound leaves.	[51]
	**Mulberry (*Morus* genus)**: Lobes develop preferentially on leaves’ outer side; many-lobed leaves retain lower temperatures; young plants have more lobed leaves.	[44,52]

**Table 2:** Sun vs. shade leaf shape parameters across multiple oak species (two-sample *t* test) ^1^ (abbreviations: SD = standard deviation, Dif. = difference, Std Err = standard error, df = degrees of freedom, CL = confidence level).

Shape Parameter	Leaf Type	Mean	SD	Dif.	t ratio	Std Err Dif.	df	*p*	95% CL Dif.	
									Lower	Upper
**Perimeter (cm)**	SUN	59	24	14	4.72	3.06	182.81	<0.0001	8	20
	SHADE	45	19							
**LDI (normalized perimeter)**	SUN	89	47	26	4.30	6.02	184.07	<0.0001	14	38
	SHADE	63	38							
**Convexity**	SUN	0.55	0.15	−0.08	−3.75	0.02	203.46	0.0002	−0.13	−0.04
	SHADE	0.64	0.17							
**Roundness**	SUN	0.60	0.08	−0.04	−3.28	0.01	186.64	0.001	−0.07	−0.02
	SHADE	0.64	0.10							

^1^ N_shade_ = 110, N_sun_ = 96; *p*-values for μ_shade_ ≠ μ_sun_ (assuming unequal variances).

**Table 3:** Sun vs. shade leaf circularity and solidity within three oak species (two-sample *t* tests) ^1^ (abbreviations: SD = standard deviation, Dif. = difference, Std Err = standard error, df = degrees of freedom, CL = confidence level).

Oak Species (Sample Size)		Parameter	Leaf Type	Mean	SD	Dif.	t Ratio	Std Err Dif.	df	*p*	95% CL Dif.	
											Lower	Upper
*Q. alba* ^2^												
N_sun_ = 21	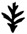	LDI	SUN	81	33	38	4.89	7.71	27.14	<0.0001	22	54
			SHADE	43	15							
N_sha_ = 26	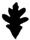	Solidity	SUN	0.68	0.12	−0.13	−4.53	0.03	28.45	<0.0001	−0.19	−0.07
			SHADE	0.82	0.06							
*Q. macrocarpa* ^2^												
N_sun_ = 11	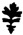	LDI	SUN	127	43	55	3.61	15.19	16.80	0.0022	23	87
			SHADE	72	28							
N_sha_ = 13	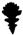	Solidity	SUN	0.75	0.04	−0.06	−4.04	0.01	16.02	0.0009	−0.09	−0.02
			SHADE	0.80	0.02							
*Q. ellipsoidalis* ^2^												
N_sun_ = 7	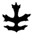	LDI	SUN	121	33	29	2.16	13.65	8.59	0.0609	−2	61
			SHADE	91	23							
N_sha_ = 16	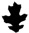	Solidity	SUN	0.46	0.06	−0.12	−4.17	0.03	11.79	0.0014	−0.19	−0.06
			SHADE	0.58	0.07							

^1^*p*-values for μ_shade_ ≠ μ_sun_ (assuming unequal variances); ^2^ leaf contours not to scale.

**Table 4:** Thermal design features and hypotheses abstracted from the leaf literature review.

BIOLOGY Plant Role Model	ABSTRACTION Shape Features, Transfer Hypotheses	APPLICATION Transfer Regime
*Viola septemloba* *Geranium sanguineum*	• **Elliptic lobation** • **Lobe elongation** *Elliptic lobes in two-dimensional exchangers enhance convection. Elliptic lobes of higher aspect ratio (elongated) enhance convection.*	SENSIBLE HEAT convective cooling
*Viburnum genus*	• **Leaf blade elongation** *Shapes of high aspect ratio enhance convection.*	
*Acer rubrum*	• **Toothed edges** • **Teeth proportions and shape** *Toothed edges, especially with proportionally large teeth, enhance vapor dissipation.*	LATENT HEAT evaporation
*Ulmus genus*	• **Hierarchically toothed edges** *Hierarchical teeth enhance vapor dissipation.*	
*Quercus genus*	• **Sinus profile in lobed shapes** *Sinuses of lobed shapes enhance orientation-independence of transfer in free convection, and inclination-independence under strong airflows.*	HEAT and MASS TRANSFER evaporative cooling
*Trevesia palmata*	• **Compoundness** • **Fenestration** *A large surface dissected into semi-distinct, space-filling parts enhances transfer.*	
*Morus genus*	• **Circular lobation** • **Convex teeth** *A hierarchical design of major obovate lobes and marginal curved teeth enhances transfer.*	

**Table 5:** Abstract geometries inspired by generic leaf morphotypes.

Geometry (i.d. and Visual)		Abstract Design Features	Relative ^1^ Perimeter	LDI	Relative ^1^ Max. Inscribed Circle Diameter	Relative ^1^ L_eff_^2^ (Effective Dimension)	Protrusion to Core Ratio	Convexity	Solidity
**C**	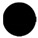	circular, control	1	13	1	1	0	1	1

**E**	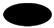	ellipse, aspect ratio and elongation	1.1	15	0.70	0.70 ^2^	0	1	1

**L**	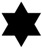	“lobes”, few and large protrusions	1.5	28	0.78	0.79	0.4	0.87	0.67

**T**	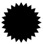	marginal teeth, many small protrusions	1.8	41	0.9	0.82	0.2	0.61	0.83


^1^ relative parameters are in relation to the control shape C; ^2^ L_eff_ computed for airflow perpendicular to the ellipse.

**Table 6:** Mean evaporation flux of leaf-inspired shape models (one-way ANOVA) (abbreviations: SD = standard deviation, Std Err = standard error, df = degrees of freedom).

Characteristic Dimension	Shape		Evap. Flux Mean ^1^ (mg/min/cm^2^)	SD	Std Err	Relative^2^ Enhancement	df	F Ratio	*p*
**D = 5 cm**	**C**	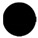	0.67	0.03	0.01	-	3	19.08	<0.0001
	**E**	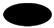	0.69	0.02	0.01	+4%			
	**L**	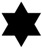	0.76	0.02	0.01	+14%			
	**T**	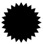	0.76	0.01	0.01	+14%			
**D = 10 cm**	**C**	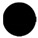	0.59	0.01	0.005	-	3	28.89	<0.0001
	**E**	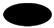	0.59	0.01	0.005	−1%			
	**L**	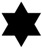	0.64	0.01	0.005	+7%			
	**T**	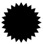	0.63	0.01	0.005	+6%			

^1^ N = 4 for each mean; ^2^ enhancement in relation to control model C.

## Supplementary Materials

The following is available online at https://www.mdpi.com/2313-7673/4/4/75/s1.
